# Serum GFAP in multiple sclerosis: correlation with disease type and MRI markers of disease severity

**DOI:** 10.1038/s41598-020-67934-2

**Published:** 2020-07-02

**Authors:** Xavier Ayrignac, Emmanuelle Le Bars, Claire Duflos, Christophe Hirtz, Aleksandra Maleska Maceski, Clarisse Carra-Dallière, Mahmoud Charif, Frédéric Pinna, Pauline Prin, Nicolas Menjot de Champfleur, Jérémy Deverdun, Tobias Kober, Bénédicte Marechal, Mario Joao Fartaria, Ricardo Corredor Jerez, Pierre Labauge, Sylvain Lehmann

**Affiliations:** 1 Département de Neurologie, CRC sclérose en Plaques, CHU Montpellier, Univ Montpellier, INSERM, 80 Av Augustin Fliche, 34295 Montpellier, France; 2 Department of Neuroradiology, Montpellier University Hospital Center, Montpellier, France; 3 I2FH, Institut D’Imagerie Fonctionnelle Humaine, Hôpital Gui de Chauliac, CHRU de Montpellier, Montpellier, France; 4 Laboratoire Charles Coulomb, CNRS UMR 5221, Université de Montpellier, Montpellier, France; 5 Economic Evaluation Unit, Centre Hospitalier Regional Universitaire de Montpellier, Montpellier, France; 6 Laboratoire de Biochimie-Protéomique Clinique, Centre Hospitalier Regional Universitaire de Montpellier, Montpellier, France; 7 IRB, Institute of Regenerative Medicine and Biotherapy IRMB – INSERM, Montpellier, France; 8 Advanced Clinical Imaging Technology, Siemens Healthcare, Lausanne, Switzerland; 9 Department of Radiology, Lausanne University Hospital, University of Lausanne, Lausanne, Switzerland; 10 LTS5, École Polytechnique, Lausanne, Switzerland

**Keywords:** Neuroimmunology, Multiple sclerosis

## Abstract

Neurofilament light chain (NfL) has been demonstrated to correlate with multiple sclerosis disease severity as well as treatment response. Nevertheless, additional serum biomarkers are still needed to better differentiate disease activity from disease progression. The aim of our study was to assess serum glial fibrillary acid protein (s-GFAP) and neurofilament light chain (s-NfL) in a cohort of 129 multiple sclerosis (MS) patients. Eighteen primary progressive multiple sclerosis (PPMS) and 111 relapsing remitting MS (RRMS) were included. We showed that these 2 biomarkers were significantly correlated with each other (R = 0.72, *p* < 0.001). Moreover, both biomarkers were higher in PPMS than in RRMS even if multivariate analysis only confirmed this difference for s-GFAP (130.3 ± 72.8 pg/ml vs 83.4 ± 41.1 pg/ml, *p* = 0.008). Finally, s-GFAP was correlated with white matter lesion load and inversely correlated with WM and GM volume. Our results seem to confirm the added value of s-GFAP in the context of multiple sclerosis.

## Introduction

Multiple sclerosis (MS) is a complex autoimmune neurological disease^1^. Despite progresses in the management of MS, reliable and easy-to-use biomarkers are needed to accurately identify patients at risk of future disease progression^2^.

The recent development of highly sensitive immunoassay platforms has enabled the measurement in the serum of several biomarkers of interest in MS. Notably, serum neurofilament light chain (s-NfL) is correlated with disease activity, treatment response, risk of disease progression and MRI markers of disease activity/severity^3–8^. Serum Glial Fibrillary Acid Protein (s-GFAP), an intermediate astrocytes cytoskeletal protein, has been only more recently shown to be higher in progressive MS than in RRMS and correlate with disability^9–11^.

Our aim was to evaluate s-GFAP value in an exploratory MS cohort and to assess its potential added value in combination with s-NfL value.

## Methods

Patients were recruited in a single-centre cross-sectional study. MS diagnosis was done retrospectively according to the 2017 McDonald criteria^12^. All the MS patients with serum sampled between January 2016 and December 2018 were included in the study. Patients had either relapsing–remitting MS (RRMS) or primary progressive MS (PPMS). The following variable were analysed: gender, age, disease duration, Expanded Disability Status Scale (EDSS), disease-modifying therapy, and existence of a clinical relapse in the past 3 months before blood sampling.

Blood samples (and CSF samples for 37 patients) were collected, and serum was stored at − 80 °C. Samples were submitted to only one freeze thaw cycle and were store less than 2 years before use. GFAP and NfL were measured using the Neurology-4-plex assay (Simoa technology, Quanterix Corporation, Lexington, MA, USA). The limit of quantification for NfL and GFAP were 0.24 pg/mL and 0.47 pg/mL, respectively. Sample results show low inter assay variation with coefficient of variation less than 12% and 5% for NfL and GFAP. Quality controls provided in the kit whit low and high concentration of NfL and GFAP also showed inter and intra assay variations, with CV lower than 10% and 4%, respectively.

MRI analysis of 50 patients was obtained for 36 patients with a 3T Skyra Siemens scanner (VE11C software version, 32 element head Coil) and for 14 patients with a 1,5T Aera Siemens scanner (VE11C software version, 32 element head coil). Only patients with no gadolinium enhancement were analysed. The acquisition protocol included a sagittal 3D Fluid-Attenuated Inversion Recovery (FLAIR) and sagittal T1-weighted magnetization-prepared rapid 3D gradient-echo (MPRAGE): 3D-T1 parameters 1,5T/3T: TR 2,400/2300 ms TE 3,47/2,98 ms, TI 1,000 /900 ms, θ = 8°/9°, BW 180/240 Hz/Px, voxel size = 1.25 × 1.25 × 1.25/1 × 1 × 1.2 mm; 3D-FLAIR parameters 1,5T/3T : TR 5,000/5000 ms TE 336/384 ms, TI 1,800/1,800 ms, Factor Turbo 222/278, BW 590/751 Hz/Px, voxel size = 1.09 × 1.09 × 1.2 mm/0.9 × 0.9 × 0.9 mm. A quality check based on image and lesion segmentation analysis was performed by two imaging experts (ELB and MJF). From this analysis 5 out of 50 cases were excluded from our dataset due to (1) missing data (ie, one of the contrasts was not acquired), (2) poor image quality due to noise or artefacts, (3) low lesion segmentation quality (inconsistent results from the automated segmentation).

The White Matter Hyperintensity load (WMHL) volume was computed on 3D-FLAIR using automated prototype software initially designed for Multiple Sclerosis lesion segmentation^13^. This method consists of two main steps: (1) pre-processing, where the images are aligned, skull-striped, corrected for bias field and intensity-normalized; and (2) lesion segmentation, performed by a supervised classifier based on k-nearest-neighbor (k-NN) algorithm that outputs a lesion probability map, ie, each voxel is labeled with a value representing the probability of containing lesion tissue^14^. Spatial constraints to estimate realistic concentration maps of healthy tissues (White Matter, Grey Matter, Cerebrospinal Fluid), and pathological brain tissue were included. These probability maps are used to directly compute lesion volumes. In addition, this algorithm proposes to automatically assess WMHL while taking into account the mixing of healthy and lesional tissue in the image voxels due to partial volume effects^15^.

Owing to their intensity on 3DT1, white matter lesions may be erroneously segmented as gray matter. Thus, areas identified as WMHL on 3DFLAIR and responsible of hypointense lesions on 3DT1 were filled by the mean global WM intensity of the patient. Resulting images were then segmented using the DARTEL approach in SPM12 (Statistical Parametric Mapping; the Wellcome Trust Center for Neuroimaging, UK). Finally, WFU pickatlas lobar template was warped to subject space to estimate the regional gray matter (GM), white matter (WM) and WMHL volumes.

Statistical analyses were performed using SAS. Biomarkers were log-transformed. After that, correlation between quantitative variables was measured using Pearson’s rho, and comparison of biomarkers between groups was tested using Student’s t-test. Disease duration was not transformed, and its correlation with biomarkers was measured using Spearman’s rho. The adjusted association between each biomarker and 1/the type of disease (PPMS or RRMS), and 2/the presence of recent relapse in patients with RRMS, was assessed using logistic regressions. We tested potential confounding of known confusion factors (age, disease duration), and selected variables with significant p-value in the multivariable model. All analyses are considered significant for *p* < 0.05.

All patients gave written informed consent. Local Ethic committee of the Montpellier University Center approved the study (2019_IRB-MTP_02-15). Our study was performed in accordance with the Current French ethical standards.

## Results

One-hundred-twenty-nine patients (97 females, 111 RRMS and 18 PPMS) were recruited. Main characteristics of the patients are disclosed in the Table 1.

**Table 1:** Characteristics of the patients.

	Whole group (n = 129)	PPMS (n = 18)	RRMS (n = 111)	*p*-value PPMS versus RRMS (raw/adjusted)	RRMS patients		*p*-value (active vs inactive RRMS
					Active (n = 18)	Inactive (n = 93)	
No. of females (%)	75.2%	77.8%	74.8%	1	50%	79,6%	0.067
Age, years (mean ± SD)	**41.5 ± 11**	**50.8 ± 7.2**	**39.9 ± 10.8**	**0.0001**	35.3 ± 9.1	37.7 ± 10.8	0.473
Age at MS onset, years (mean ± SD)	**34.3 ± 11.2**	**46.9 ± 7.3**	**32.3 ± 10.5**	** < 0.0001**	33,6 ± 10	32,1 ± 10.5	0.603
Disease duration, months (mean ± SD)	80 ± 86	42 ± 15	86 ± 90	0.28	**20 ± 62**	**98 ± 60**	** < 0.0001**
EDSS, median, IQR	**1.75 (0.0; 3.0)**	**3.5 (2.0; 6.0)**	**1.0 (0.0; 3.0)**	** < 0.0001**	1.5 (1.0 ; 2.0)	1.0 (0.0 ; 3.0)	0.736
Current DMT (%)	**41.9%**	**None**	**48.7%**	**0.0001**	**5,6%**	**57%**	**0.0001**
**Serum biomarkers (pg/ml)**							
GFAP (mean ± SD)	**89.9 ± 49**	**130.3 ± 72.8**	**83.4 ± 41.1**	**0.0032**	**63,3 ± 21 .7**	**87,3 ± 43**	**0.015**
Log GFAP	**4.38 ± 0.48**	**4.73 ± 0.55**	**4.32 ± 0.44**	0.0007/0.008*	**4.08 ± 0.41**	**4.37 ± 0.43**	**0.015**
NfL	**9.93 ± 5.06**	**13.63 ± 6.60**	**9.33 ± 4.52**	**0.0016**	8,95 ± 5.09	9.40 ± 4,43	0.64
Log NfL	**2.18 ± 0.47**	**2.51 ± 0.48**	**2.13 ± 0.45**	**0.0014/0.40†**	2.06 ± 0.53	2.15 ± 0.43	0.45
**MRI volume (n)**	(n = 45)	(n = 8)	(n = 37)				
T2 lesion load	14.03 ± 13.00	20.19 ± 17.40	12.70 ± 11.72	0.18	NA	NA	NA
WM vol	424.03 ± 55.24	415.11 ± 50.96	425.96 ± 56.60	0.60	NA	NA	NA
GM vol	**662.95 ± 82.72**	**600.04 ± 46.8**	**679.64 ± 76.70**	**0.01**	NA	NA	NA
Cortical GM vol	**645.5 ± 76.04**	**581.9 ± 44.9**	**659.3 ± 74.7**	**0.005**	NA	NA	NA

*PPMS* Primary progressive multiple sclerosis; *RRMS* relapsing remitting multiple sclerosis; *EDSS* expanded disability status scale; *DMT* disease modifying therapy; *GFAP* glial fibrillary acid protein; *NfL* neurofilament light chain; *WM* white matter; *GM* grey matter; *NA* not available.

*Covariables were age and disease duration. **†**Covariables were log GFAP, age and disease duration.

**Figure 1:**
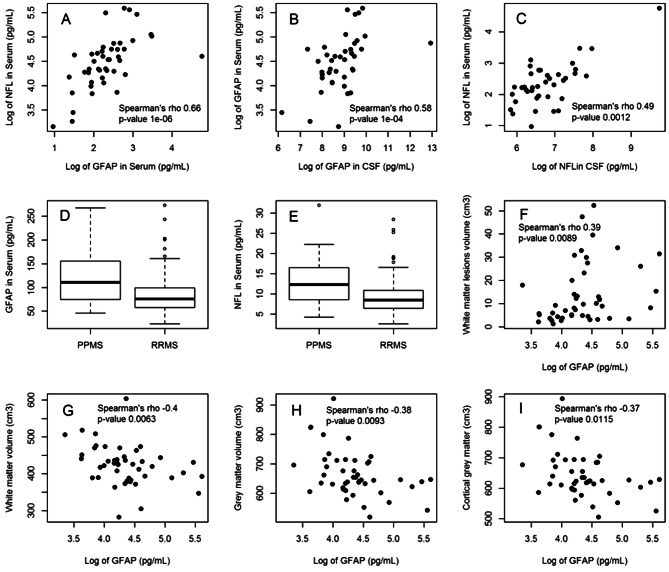
(**A**) S-GFAP and s-NfL levels are correlated in MS patients (r = 0.66, *p* = 1e−06). Serum and CSF GFAP (**B**) and NfL (**C**) were correlated (r = 0.58, *p* = 1e−04 and r = 0.48, *p* = 0.0012). s-GFAP (**D**) and s-NfL (**E**) levels are higher in PPMS than in RRMS (respectively 130.3 ± 72.8 vs 83.4 ± 41.1 pg/ml and 13.63 pg/ml 6.60 vs. 9.33 pg/ml 4.52 pg/ml). S-GFAP is correlated with white matter lesions volume (F, r = 0.39, *p* = 0.0093) and is negatively correlated with white matter (G, r = − 0.4, *p* = 0.063), grey matter (H, r =  − 0.38, *p* = 0.0093) and cortical grey matter (I, r = − 0.37, *p* = 0.0115) volumes.

S-GFAP and s-NfL levels were correlated with each other (Fig. 1A) and, for each biomarker, serum levels correlated with CSF levels (respectively R = 0.56 (95% IC: 0.28–0.75), *p* = 0.0004 and R = 0.51 (95% IC 0.22–0.72), *p* = 0.0016, Fig. 1B,C). Whereas s-GFAP was also correlated with age, disease duration and disease type, s-NfL was correlated with age and disease type. After adjustment for age, the levels of the 2 biomarkers were not independently correlated with EDSS level (either in RRMS group or in PPMS group) and treatment (not shown).

PPMS patients had higher s-GFAP and s-NfL levels than RRMS patients. After multivariate analysis including age and disease duration, s-GFAP (but not s-NfL) remained significantly higher in PPMS (Fig. 1D,E).

In the RRMS group, the presence of a recent relapse was correlated with lower s-GFAP but this association was not significant after adjustement for disease duration. S-NfL were similar in active and inactive patients.

Lesion load and brain volume were analysed in 45 patients (37 RRMS). s-GFAP level correlated with white matter lesion load and inversely correlated with WM, GM and cortical GM volumes (Table 1, Fig. 1F–I)). s-NfL level was not correlated with MRI parameters.

## Discussion

Our results, in line with 3 recent studies, suggest that s-GFAP is an additional serum biomarker of interest in the context of MS^9–11^. Indeed, we confirm, that s-GFAP and s-NfL, are higher in PPMS than in RRMS. Even if some studies (including CSF studies) disclosed contradictory results, s-NfL has been repeatedly demonstrated to be higher in PPMS than in stable RRMS patients^3–6^. We suggest that, partly due to the small size of our cohort, our study was underpowered to demonstrate a significant difference after adjustment. Moreover, the absence of correlation between biomarkers and EDSS was probably due to the relative benign disease severity of our cohort since most of the patients had RRMS with low EDSS (median EDSS value: 2.75, 83% with EDSS ≤ 3).

To date, mainly due to conflicting published results, it is unclear whether s-GFAP values are increased or decreased during relapses^10,16–20^. Our study, did not evidenced any significant difference of s-GFAP values in patients with and without recent relapses suggesting that larger cohorts are needed to identify any significant and meaningful difference. Moreover, were not able to confirm previous studies disclosing higher s-NfL level in patients with recent relapse. This was probably due to the small size of our cohort since only 18 patients had recent relapse. This was also probably caused by the 3-months period used to define a recent relapse since it has been shown that s-NfL steadily decrease from a pic at the time of a relapse to baseline levels approximately 3 months after the relapse^21^.

To date, apart from retrospective analysis of randomized control trials, only few studies have correlated serum biomarkers and MRI data in MS^3–6^. Most of these studies used semi-quantitative MRI assessment and/or had lower sample size than our study. Only one recent study disclosed an association between s-GFAP (T1 and T2) lesion load and cortical GM atrophy^10^. With respect to s-NfL, 4 studies mainly disclosed a positive association with T2 lesion load^3–6^. Our analysis in 45 inactive patients showed that s-GFAP is correlated with T2 lesions load and (negatively) with WM and GM volumes. S-NfL were not correlated with any of the MRI measurements. These discrepancies are probably explained by methodological issues and biases underlining that larger cohorts are needed to identify accurate correlations between serum biomarkers and MRI measures.

Our study has some limitations, notably its retrospective design as well as the small size and the relative benign disease severity of our cohort that limited our statistical power to identify significant associations.

Nevertheless, we suggest that s-GFAP is a potentially interesting marker to distinguish PPMS and RRMS. Whether or not it can be useful to distinguish, in combination with s-NfL, disease activity from disease progression remains to be further assessed in larger independent cohorts.
